# Material wealth in 3D: Mapping multiple paths to prosperity in low- and middle- income countries

**DOI:** 10.1371/journal.pone.0184616

**Published:** 2017-09-08

**Authors:** Daniel J. Hruschka, Craig Hadley, Joseph Hackman

**Affiliations:** 1 School of Human Evolution and Social Change, Arizona State University, Tempe, Arizona, United States of America; 2 Anthropology Department, Emory University, Atlanta, Georgia, United States of America; Universidad Veracruzana, MEXICO

## Abstract

Material wealth is a key factor shaping human development and well-being. Every year, hundreds of studies in social science and policy fields assess material wealth in low- and middle-income countries assuming that there is a single dimension by which households can move from poverty to prosperity. However, a one-dimensional model may miss important kinds of prosperity, particularly in countries where traditional subsistence-based livelihoods coexist with modern cash economies. Using multiple correspondence analysis to analyze representative household data from six countries—Nepal, Bangladesh, Ethiopia, Kenya, Tanzania and Guatemala—across three world regions, we identify a number of independent dimension of wealth, each with a clear link to locally relevant pathways to success in cash and agricultural economies. In all cases, the first dimension identified by this approach replicates standard one-dimensional estimates and captures success in cash economies. The novel dimensions we identify reflect success in different agricultural sectors and are independently associated with key benchmarks of food security and human growth, such as adult body mass index and child height. The multidimensional models of wealth we describe here provide new opportunities for examining the causes and consequences of wealth inequality that go beyond success in cash economies, for tracing the emergence of hybrid pathways to prosperity, and for assessing how these different pathways to economic success carry different health risks and social opportunities.

## Introduction

Material wealth is a key factor shaping human behavior, psychology, and development and has shown well-established relationships with a wide range of behaviors and outcomes, including fertility [1–4], dietary choices [5,6], food security [7,8], physical growth in children and adults [9–14], investment in education [15], cognitive function [16,17], and differential participation in community helping and social exchange [7,18–20]. Researchers have proposed a number of ways that material wealth can influence individual development and well-being, including improved nutrition, better access to infrastructure that prevents disease (e.g. clean water), the ability to seek medical care for illnesses and emergencies, and the capacity to buffer temporary losses of income [21,22]. Material wealth also provides opportunities for educational and occupational attainment which in turn can shape development and well-being in a number of ways [21]. For these reasons, social scientists have long been interested in the diverse ways that humans seek, create, maintain, and use material wealth, how wealth inequalities arise and persist, and how inequalities shape human behavior and development [11,23–36].

Social scientists use several approaches to assess economic achievement. In high-income countries, economists, sociologists, and demographers have traditionally focused on income and expenditures [21,37]. However, the measurement of such flows poses challenges in low- and middle-income countries related to cost, reliability, validity and stability [37,38]. For these reasons, social scientists working in low- and middle-income countries have often relied instead on asset-based assessments of material wealth to capture the economic resources available to individuals and households [22,37,39,40]. Particularly in the last two decades there has been striking growth in the use of asset-based approaches relying on: (a) ownership of goods, such as televisions, bicycles, and telephones, (b) housing construction such as wall and floor type, (c) ownership of land, cattle, and other forms of capital, (d) and access to basic services, such as electricity and clean water [37]. These measures of material wealth are intended to capture the long-run economic capacity of households, and thus are conceptually distinct from other measures of socioeconomic status, such as education or occupational status [21,39,41].

A tacit assumption underlying nearly all asset-based wealth estimates is that there is a single dimensions of material wealth along which households can be ranked from wealthiest to poorest. This is estimated as a single weighted sum of the assets, housing, and services to which household has access. Every year, hundreds of studies in the social sciences and development, many in high profile journals, use such one-dimensional estimates of wealth for a variety of purposes—to assess equity in access to health and family planning services [42,43], to determine economic disparities in health and educational outcomes [44,45], to screen high-risk families [46], to estimate economic growth [47], to examine how social inequality is related to social unrest and other outcomes [48], and to benchmark other measures of economic capacity [49,50]. In nearly all cases, these one-dimensional measures are interpreted generically as “wealth” and reflect a tacit consensus that there is only one way to become wealthy in these countries.

These one-dimensional indices have permitted researchers to examine health disparities and assess economic growth in novel ways that are important for both social science theory and policy analysis. However, in-depth ethnographic observations suggest that they also potentially mask other local paths to accumulating material wealth [26,51,52]. A common distinction is made between success in agricultural economies versus the accumulation of market-based assets through cash economies [44,53–55]. Success along these different dimensions may create distinct opportunities, constraints, challenges, and risks for households, that would be confounded with a single-dimensional estimate of wealth [52]. For example, disentangling achievement in the agricultural economy from achievement in the cash economy is important for human capital theories of fertility transitions that propose parents will make very different investments in fertility and offspring when they are predominantly engaged in agricultural production versus professional wage employment [4,55,56]. Multidimensional models of material wealth can also assist efforts to understand how different kinds of economic activity expose individuals to different health risks. For example, for the last decade, researchers have puzzled over why increasing wealth in some sub-Saharan countries is associated with increased risk of testing positive for HIV. One hypothesis for this finding is that common wealth measures are actually assessing engagement with one kind of economy—the urban cash economy—which could bring additional risk of HIV transmission from more far-flung social interactions [44]. Similarly, multidimensional models of material wealth can allow researchers and policymakers to explore how success in different kinds of livelihoods is related to resilience and vulnerability in the face of shocks or shortages, such as famine or climate change events [57,58]. Though potentially important, additional dimensions of material wealth are largely ignored in the current measurement of material wealth. Thus, it is not clear how many additional dimensions are required to capture these alternate pathways or how achievement along these alternate paths matter for key outcomes of interest. In addition to the examples described here, methods for estimating multiple dimensions of wealth could potentially expand our understanding of inequality and how it relates to a wide range of welfare indicators, including food security, water security, and mental well-being.

In the last decade, demographic and health surveys worldwide have collected a much greater diversity of data on material assets—including fine-grained information on livestock, land and agricultural assets—permitting us to explore such alternative pathways to material wealth. In this paper, we use this new data to tackle a number of questions. First, can we reliably estimate independent dimensions of material wealth from these assets that go beyond a single dimension? Second, if additional reliable dimensions exist, do they have meaningful local interpretations in terms of additional paths to prosperity? Third, as a test of the construct validity of these new dimensions, are they independently associated with key nutritional indicators—household food security, adult body mass index (BMI), and child height-for-age—that have well-established associations with increasing economic resources in low-income countries?

We investigate these questions by analyzing household data on asset ownership from nationally representative surveys conducted in six low-income countries that span three world regions—sub-Saharan Africa, South Asia, and Central America. Crucially, these datasets provided rich data on household access to material goods and services across a range of domains, including consumer goods, access to basic services, housing construction, and land and livestock ownership.

To estimate multiple dimensions of wealth from the ownership of assets, we use multiple correspondence analysis (MCA) [59]. The procedure represents a “cloud of households” in a multidimensional livelihood space where distances between households are based on differences in ownership of goods and services (e.g. owning a TV, owning land, access to electricity) [60]. MCA then identifies the orthogonal dimensions in this cloud that successively capture the most variation in ownership of of goods and services. These derived dimensions can then be used as composite measures of material wealth along different dimensions. This approach parallels earlier work by sociologists determining different pathways to the accumulation of material, social, and cultural capital [61]. The procedure also has deep commonalities with principal components analysis (PCA), which is frequently used to estimate wealth scores from asset-based data [37,60]. While PCA can also estimate multiple orthogonal dimensions from such data, MCA is specifically designed for the kinds of nominal categorical data that is usually collected for asset ownership [62]. Thus, MCA provides an appropriate and useful tool for estimatingorthogonal dimensions of wealth accumulation from asset-based data.

With each household assigned values along these dimensions of wealth, we can map individual households (and the average location of groups of households holding a specific asset) in a multi-dimensional livelihood space where households that are closer to each other are more similar in their household assets. To examine the construct validity of these dimensions and the value added by additional dimensions, we also compare household’s values on these different dimensions with other benchmarks of human achievement, such as food security, adult BMI, and child height. These analyses show that: (1) at least two reliable dimensions of wealth exist in all six countries, (2) these dimensions have clear interpretations in terms of achievement along different pathways to prosperity, and (3) they are independently associated with improved food security and indicators of physical growth in both children and adults. Thus, these additional dimensions are not only reliable, but they also provide supplementary information about material achievement that is independently related to key benchmarks of human well-being.

## Methods

### Data

We analyse data from five Demographic and Health Surveys and one Living Standards Measurement Survey (LSMS) which represent a much larger set of nationally representative surveys collecting relatively standardized information on households and individuals in more than 70 low- and middle-income countries. Given the challenges of harmonizing agricultural data across these diverse datasets, we focused the current analyses on six countries—four with which the authors have extensive ethnographic experience and knowledge about local livelihoods (Bangladesh 2011, Ethiopia 2010, Tanzania 2015, Guatemala 2000) and two neighboring countries for which a DHS also included food security questions (Nepal 2011 and Kenya 2014). We based analyses on the most recent survey that contained all necessary variables. The surveys include data on: (1) a range of household assets and access to basic services (see S1 Table for full list by country), (3) the height and weight of adult women, and (4) the height of children. They also include additional key variables—household sampling weights, woman’s age and pregnancy status, child’s age, and household rural-urban designation. Five of these datasets and survey collection protocols are freely available through Measure DHS (measuredhs.com). The sixth dataset for Guatemala is the 2000 Living Standards Measurement Survey and is freely available through the World Bank (http://iresearch.worldbank.org/lsms/lsmssurveyFinder.htm).

### Samples

For multiple correspondence analysis of household wealth, we include all available households (Bangladesh n = 17300, Nepal n = 19533, Kenya n = 17409, Ethiopia n = 16702, Tanzania = 12563, Guatemala n = 7276). Physical growth is a sensitive indicator of increasing economic resources among individuals living on less than $10 US per day, a threshold used by policymakers to identify populations in a state of vulnerability [63]. However, it shows varying relationships with wealth among populations living above this threshold [13,64]. In five of the six countries, nearly all individuals were living under $10 US per day (Ethiopia = 98.5%, Kenya = 93.7%, Nepal = 96.6%, Tanzania = 98.2%, Bangladesh = 97.3% (Povcal.net)). By contrast, approximately 30% of Guatemalan households were above this threshold. To focus on individuals where physical growth would be a sensitive indicator of economic resources, we exclude the top 25% of households in the Guatemala sample ranked by total estimated per-capita household consumption. For analysis of physical growth, we include 20–49 year old, non-pregnant women (Bangladesh n = 26815, Nepal n = 10344, Kenya n = 15340, Ethiopia n = 13638, Tanzania n = 7801, Guatemala n = 3866) and 0–24 month old boys and girls (Bangladesh n = 1527, Nepal n = 2499, Kenya n = 5123, Ethiopia n = 4283, Tanzania n = 1958, Guatemala n = 1863). The first women and child (if any) recorded in a given household were used for analyses. Household food security data were available for Kenya (n = 17409), Nepal (n = 10826), and Tanzania (n = 12563).

### Variables

#### Household construction, assets, and basic services

For each country, we examined a set of variables recording household construction, assets and access to basic services. These include source of drinking water, toilet type, wall, roof and floor material, cooking fuel, electrical access, and ownership of livestock, land, and a range of assets, vehicles and consumer goods (see S2 Table). Variables with more than two nominal categories—drinking water source, toilet type, housing construction, and cooking fuel—were dummy coded as a series of dichotomous variables. Count variables for ownership of livestock and agricultural land were recoded into dummy variables for different levels of ownership. This resulted in a total of 250 unique dichotomous variables across the set of 6 surveys—16 for water source, 14 for toilet type, 10 for floor type, 19 for wall type, 16 for roof type, 13 for cooking fuel type, 61 for livestock ownership, 13 for land ownership, 44 for household goods, 8 vehicles, 21 for agricultural equipment, installations, and items cultivated and 15 variables for ownership of one’s home, a business and possession of a bank account. The number of variables available for each of the six countries ranged from 115 to 159 (frequencies of each variable in S2 Table). For subsequent comparison with dimensions derived from correspondence analyses, livestock counts and land in hectares were also log-transformed (ln(count+1)).

#### Physical growth

In each of the country samples, height and weight measures were taken by trained DHS and LSMS technicians. Body mass index of adults was calculated as weight (kg)/height (m)^2^, and cases with values below 10 or above 80 were excluded. Height-for-age Z-scores for children were based on current World Health Organization reference distributions, except in the case of Ethiopia for which CDC reference distributions were available [65]. Cases with absolute Z-scores greater than 6 were excluded from analyses [65].

#### Food security

The Kenya 2014, Nepal 2011, and Tanzania 2015 surveys asked households about food security. For Kenya and Nepal, we constructed a scale from three question that were roughly comparable across the two surveys—relying on less preferred foods, reducing the number of meals eaten, and reducing the portion size as responses to insufficient food. For Tanzania, we used the following questions—problem with meeting food needs, finding any kind of food, sleeping at night hungry due to no food, and going the whole day and night without eating The scales had acceptable internal reliability (Cronbach’s Alpha = 0.88, 0.79, 0.88 respectively). We Z-transformed the scales so that effect sizes are interpreted in standard deviations, and a positive value means increasing food security.

#### Anchors for interpreting wealth dimensions

A number of variables were used to anchor and determine the direction of dimensions derived from MCA. These include urban/rural residence, counts of each type of commonly owned livestock, amount of agricultural land owned, education of household head (no school, primary, secondary, and higher education), and whether the household had a bank account or owned a TV.

#### Covariates

When assessing the relationship of wealth dimensions with key indicators of physical growth and nutrition, age was included in analyses as a categorical variable—20–29 y, 30–39 y, and 40–49 y for women and 0-6m, 7-12m, 13-18m, 19-24m for children—to deal with potential non-linearity of age effects. Urban/rural residence and child’s sex were also included in analyses. We also controlled for educational status of the respondent (adult BMI analysis), of the mother (child HAZ analysis), or of the first female listed in the household (food security analysis) as a categorical variable (no education, primary, secondary, higher).

### Analysis

To identify independent dimensions of variation in material wealth, we applied a multiple correspondence analysis to a household-by-variable matrix for each of the six countries [59]. The variables were dichotomous asset measures described in the supplementary materials (S2 Table). Each household was then assigned a value on each of the estimated dimensions. We estimated 3 dimensions and considered only dimensions that had acceptable internal reliabilities (Cronbach’s alpha > 0.70). Each household was then assigned a coordinate along each of the independent dimensions. Based on these coordinates, one can represent the households in multi-dimensional space. For a given asset, one can also calculate the average location in the multidimensional space (i.e. centroid) of all households that own that asset or have a specific variable value (e.g. household head with secondary education). Dimensions were scaled to have a standard deviation of 1.

It is important to note that a positive value on an estimated dimension does not have an inherent meaning, and the direction of the dimension must be determined using relevant anchors. When there is a clear directional interpretation of a dimension in terms of material wealth accumulation, we set the sign of the dimension so that greater positive values indicate greater accumulation. When there is no clear directional interpretation, we retain the original signs.

Using the first two dimensions identified by MCA, we plotted the location of households in a 2-dimensional space along with a number of anchors—the centroids (or mean locations in the multidimensional space) of rural and urban households, the various educational attainments of household heads, ownership of varying quantities of land and livestock as well as TV ownership and households not having access to a toilet. We further assessed the bivariate correlations of each of the dimensions with the presence of key anchor assets and with livestock and hectare counts.

To assess the independent association of each of these dimensions with the physical growth and nutrition of household members, we conducted a regression with each of three outcomes—household food security, adult female BMI, child height-for-age—and all regressions included dummy coded variables for education and urban/rural residence. The adult BMI regression included the main effect of age along with an interaction of age with each of the dimensions of wealth and with rural residence. The child regressions included child’s sex and a main effect of child’s age. All analyses were conducted using household weights, and correlational and regression analyses were conducted accounting for complex sample design in SPSS Complex Samples.

## Results

In results, we first describe the wealth dimensions identified in the six countries using MCA and how these dimensions are systematically correlated with key assets. Second, we examine how achievement along these different dimensions is associated with key benchmarks of human growth and nutrition, and the degree to which these additional dimensions are independently associated with growth and nutrition.

### Dimensions of variation in livelihood space and their relationship to key assets

In all six countries, MCA identified at least 2 dimensions with sufficient internal reliabilities to be analyzed (α > 0.70). As would be expected, the first dimensions nearly perfectly correlated with the standard wealth index factor score derived for DHS and LSMS using a related procedure—Principal Components Analysis (r > 0.98, p < 0.001). This first factor accounted for 7.0% to 9.6% of the variance in the dichotomous wealth indicators (Table 1). Across all countries, the second factor accounted for an additional 2.7% and 3.9% of the variance in wealth indicators (Table 1)—equivalent to one-third to one-half of the variance accounted for by the first dimensions depending on the country. Three countries also had a reliable third dimension (Nepal, Kenya and Guatemala) accounting for an additional 2.5% to 2.8% of the variance in wealth indicators.

**Table 1:** Wealth dimensions and their bivariate association with key variables. α = Cronbach’s alpha, %V = % variance explained. All effect sizes significant at 0.0005 level, unless note with ^a^.

Dim	Country	α	%V	Urban	School	Bank Acct	TV	Cows	Chickens	Farmland(Ha)
1	Nepal	0.92	7.8	0.49	0.50	0.35	0.66	-0.45	-0.35	0.07
	Bangladesh	0.90	7.9	0.64	0.49	0.51	0.62	-0.29	-0.36	0.15
	Kenya	0.90	7.0	0.72	0.52	0.27	0.55	-0.08	-0.36	-0.24
	Ethiopia	0.90	7.6	0.78	0.55	0.46	0.77	-0.51	-0.36	-0.53
	Tanzania	0.90	7.5	0.71	0.54	0.51	0.73	-0.03 ^a^	-0.24	-0.44
	Guatemala	0.94	9.6	0.63	0.64	0.55	0.65	-0.13	-0.49	-0.32
2	Nepal	0.79	3.2	-0.07	0.13	0.32	0.24	0.35	0.55	0.33
	Bangladesh	0.73	3.1	-0.21	0.24	0.33	0.32	0.54	0.50	0.47
	Kenya	0.76	2.9	0.02 ^a^	0.17	0.17	0.33	0.30	0.45	0.45
	Ethiopia	0.71	2.7	0.08	0.05	0.18	0.14	0.55	0.39	0.36
	Tanzania	0.79	3.7	-0.03 ^a^	0.12	0.21	0.22	0.35	0.46	0.34
	Guatemala	0.85	3.9	-0.11	0.13	0.25	0.06	0.43	0.35	0.44

**Fig 1:**
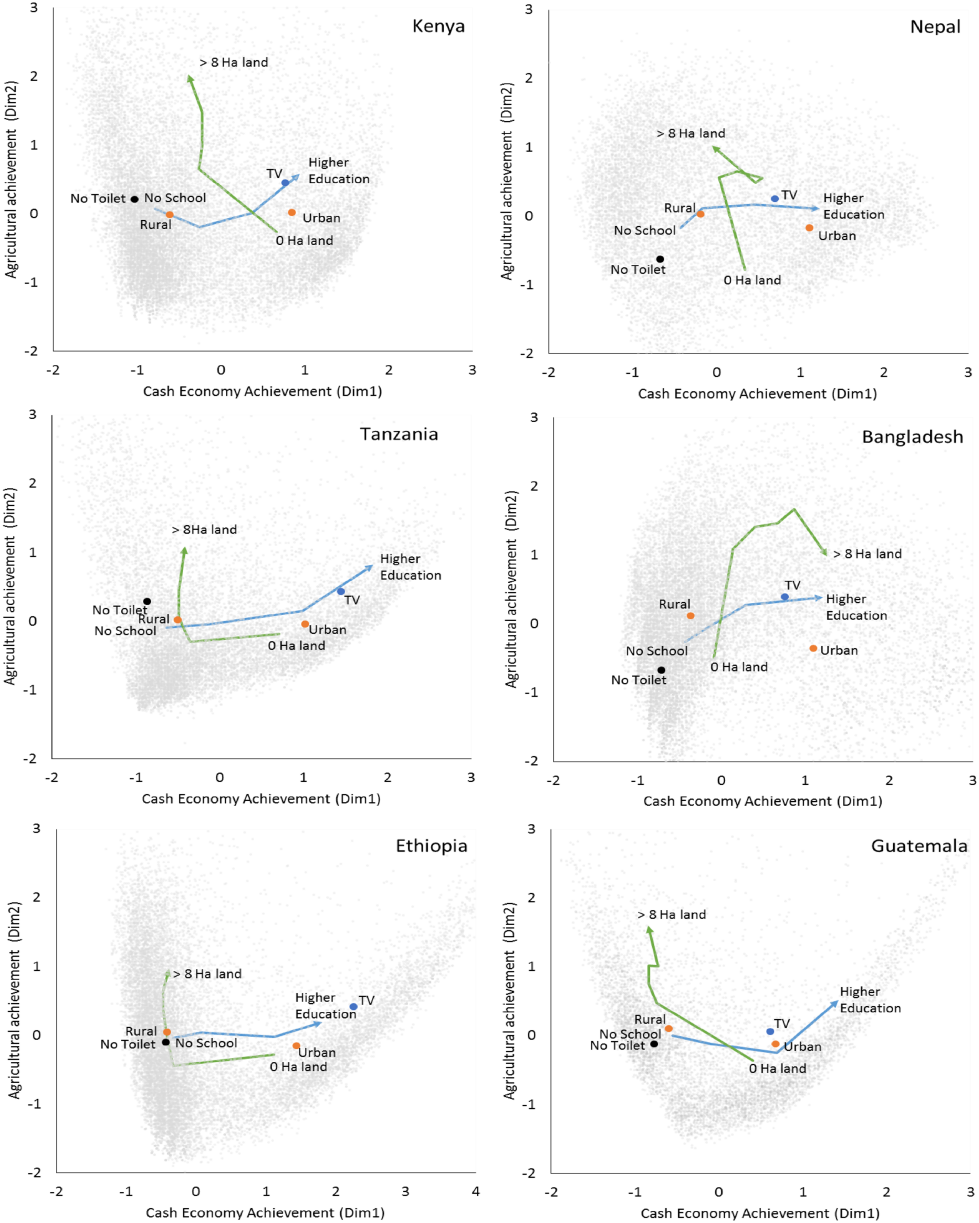
Households from six countries mapped on the first two wealth dimensions. Gray dots = households (HH). Axes = two dimensions estimated by multiple correspondence analysis. Orange dots = centroids for rural and urban HH. Blue dot = centroid for HH with televisions. Black dot = centroid for HH without toilet facilities. Green arrow = change in centroid from HH owning no land to > 8 Hectares of agricultural land (Ha). Blue arrow = change in centroid for HH head having no education to higher education.

Fig 1 illustrates households from the six countries mapped onto the first two dimensions. Anchoring centroids illustrate how households with different kinds of assets and achievement are distributed in the space. For example, the average household with no toilet facility is located in the left hand corners, usually to the lower left. Moving up along dimension 2 from the lower corner is associated with increasing ownership of agricultural land. Meanwhile, moving right along dimension 1 is associated with increasing urban residence, increasing education, and ownership of expensive consumer goods, such as televisions.

Bivariate associations in Table 1 illustrate the magnitude of correlations between each of the first two dimensions and a number of key variables. Notably, urban residence, education, and owning a TV are usually associated more strongly with dimension 1 than dimension 2 (Table 1). Meanwhile, dimension 2 is positively associated with agricultural accumulation (e.g., number of cows, chickens and hectares owned), but dimension 1 shows much weaker and usually negative associations with these same agricultural variables. Based on the strong associations of dimension 1 with greater formal education, greater possession of expensive consumer goods and greater likelihood of urban residence, we interpret it as reflecting achievement in the cash economy. Based on dimension 2’s consistently positive associations with greater ownership of animals and land, we interpret it as reflecting achievement in the agricultural economy.

Across countries, both the first and second dimensions were positively associated, to varying degrees, with having a bank account, having more education, and having a TV (Table 1). Thus, these forms of ownership and human capital appear to be common to different dimensions of material prosperity, and may be useful for anchoring dimensions across a range of contexts. However, in many cases, it is important to note that the same asset or variable can carry very different meanings between and within countries. For example, in all six countries owning a TV is much more strongly associated with achievement along the first dimension (mean correlation = 0.66) than along the second dimension (mean correlation = 0.22). For other assets, the direction of the association can even change depending on the country or kind of wealth dimension considered. For example, using water from a tubewell is positively associated with agricultural achievement in Bangladesh (dim 2, r = 0.30), but negatively associated with it in Nepal (dim 2, r = -0.47). Similarly, owning a bicycle indicates greater achievement in the agricultural economies of Bangladesh (r = 0.41, dim 2), but of lesser achievement in Nepal (r = -0.54, dim 2). This illustrates the importance of identifying locally meaningful assets for anchoring the dimensions.

**Fig 2:**
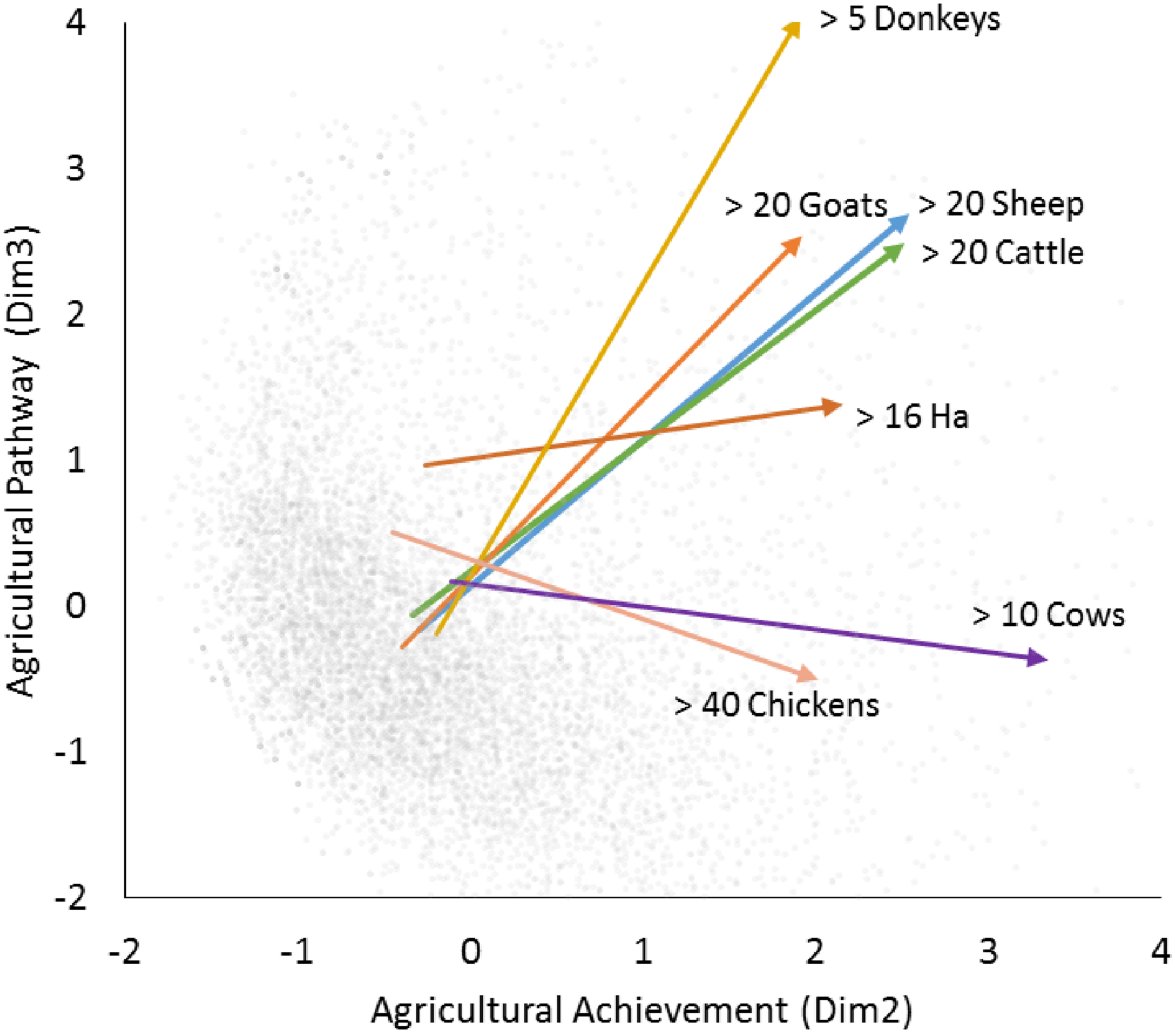
Rural Kenyan households (n = 10764) mapped along two dimensions of agricultural wealth and livelihood. Dim2 = 2^nd^ MCA dimension, Dim3 = 3^rd^ MCA dimension. Individual gray dot = household. Arrow starts = centroid of households without the specified animal. Arrow end = centroid of households having achieved a certain number of animals.

Because of the large number of households and the diversity of asset variables considered in these surveys, MCA also identified reliable third dimensions in three of the countries—Nepal, Kenya, and Guatemala (Cronbach’s α = 0.74, 0.75, 0.76, % of variance = 2.6%, 2.8%, 2.5%). These third dimensions appear to capture variation in ecological zones and related variation in housing characteristics and agricultural pursuits. For example, Fig 2 illustrates how dimension 3 in Kenya captures variation between households in terms of the kinds of livestock they have accumulated (Fig 2). While increasing values on dimension 2 reflect accumulation of a wide range of agricultural goods (e.g. land, livestock and chickens), increases along dimension 3 reflect accumulation of livestock common to semi-arid lowlands (e.g. goats r = 0.37, sheep r = 0.28, and donkeys r = 0.41, p < 0.001) but declines in other livestock more common in highland areas, such as cows and chickens (cows r = -0.35, chickens r = -0.32, p < 0.001) (S1 Fig). This variation in agricultural livelihoods is associated with ecological differences, with high values on dimension 3 concentrated in semi-arid lowland areas (Northeastern mean for dim 3 = 1.9, Coast mean for dim 3 = 0.5) and lower values on dimension 3 concentrated in highland areas (Western mean = -0.6, Central mean = -0.4, Nyanza mean = -0.3). In this case, it is not possible to assign a direction to the third dimension as it captures the style of agricultural accumulation common in certain regions rather than the total quantity of resources accumulated. Similarly, in Nepal, increases on dimension 3 are strongly associated with variables related to different ecological zones, such as higher altitudes (r = 0.67, p < 0.001) and more ownership of stone wall houses (r = 0.52, p < 0.0001), and less ownership of waterfowl (r = -0.27) and mud wall houses (see SM for more detail). (see S1 Fig for descriptions of dimension 3 in Nepal and Guatemala which also do not have a clear directional interpretation). Thus, although the first two directions have clear directional interpretations in terms of the accumulation of wealth in agricultural and cash-based economies, the third dimension when it exists, appears to capture country-specific differences in agricultural livelihoods and ecological zones.

### Validating wealth dimensions with food security and physical growth

**Fig 3:**
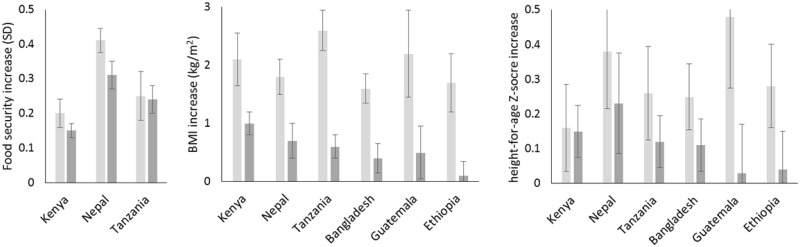
Associated increase of food security (a), adult BMI (b), and child height-for-age Z-score (c) for each standard deviation increase in cash economy (Dimension 1, light gray bar) and agricultural achievement (Dimension 2, dark gray bar). Error bars are 95% CI. Adjusted for age, education, and urban residence.

Although the first two dimensions of economic achievement are associated with two different kinds of assets, we expect that, if these dimensions truly reflect improving economic conditions, that achievement along both of these dimensions should be associated with basic nutritional improvements—proxied here by greater food security as well as increased adult fat deposition and increased child growth (Fig 3). In the three countries with data on household food security (Kenya, Tanzania and Nepal), increases along both the first and second dimensions are associated with increasing food security. Women’s BMI show similar associations in five of the six countries, and child’s HAZ in four of the countries. However, in Ethiopia, neither female BMI nor child height-for-age Z-scores significantly increase with larger values on the agricultural dimension (dimension 2).

There are clear expectations about how increases along the first two dimensions should relate to physical growth and nutrition. However, increases along the third dimensions identified in Kenya, Nepal, and Guatemala (which reflect something like type of agricultural economy or ecological zone) do not have clear directional interpretations in terms of accumulation. Thus, it is not possible to come up with clear a priori predictions about how changing values on the third dimension should be associated with nutrition and growth. However, it may still be of interest to assess whether residing at either end of the third dimension, indicating specific kinds of agricultural accumulation, is related to food security and growth. Indeed, we find that higher values on dimension 3 in Kenya are negatively associated with food security (-0.16 (-0.18,-0.13), adult BMI (-1.0 (-1.2,-0.8)) and to a lesser extent with child HAZ (-0.07 (-0.15,0.01)). This indicates agricultural accumulation common to Kenya’s semi-arid lowlands is associated with substantially worse nutrition and growth than the agricultural accumulation common to the highland regions. In Nepal, increases on dimension 3 are strongly associated with higher altitudes (r = 0.67, p < 0.001) reflecting high versus low altitude agricultural pursuits (see SM for more detail). Higher values on dimension 3 in Nepal are also positively associated with adult BMI (0.3 (0.1,0.6), and to a lesser extent food security (0.09 (0.04, 0.13), but not child HAZ (0.0, (-0.15,0.15)). By contrast, in Guatemala, increasing values on dimension 3 show no association with adult BMI (0.1, (-0.4, 0.5)) or child HAZ (0.06, (-0.06, 0.19)). Thus, in only two of the three countries where a third dimension was identified did variation along that dimension correlate nutrition and growth. Moreover, the effects were much stronger in Kenya than they were in Nepal. In those two situations, more research is needed to determine if this reflects differential risks from varying nutritional or disease ecologies, or rather underlying ethnic differences in body build [66].

## Discussion

One-dimensional models of material wealth have provided an important foundation for researchers to pursue a wide range of questions about inequality, deprivation and well-being in low income settings. However, our findings from six countries across three world regions also show that one-dimensional models capture only one kind of advancement—success in cash economies correlated with increasing education, ownership of costly consumer goods, and increasing urban residence. Consequently, they miss other avenues for advancement—most notably success in agricultural economies—that are also associated with the health and well-being of populations. Using data on agricultural assets collected in the last decade by demographic and health surveys, we estimate at least two and sometimes three independent and reliable dimensions of material achievement that capture these alternative pathways. The additional second dimensions have clear interpretations as success in the agricultural economy, including increasing ownership of common livestock, such as cows, chickens, goats and sheep, and increasing likelihood of owning agricultural land. Importantly, these seconddimensions are also independently associated with substantially greater food security as well as adult and child growth, indicating that they are not only reliable, but also biologically meaningful, measures of economic success independent of success in the cash economy. In three countries, we also identified third dimensions which reflect different kinds of agricultural livelihood specific to each country (e.g. lowland versus highland agropastoralism). In some countries this third dimension captured differences in livelihoods, such as variation between semi-arid lowland herding and highland agriculture in Kenya, that also had potentially important relationships with physical growth and food security.

A key benefit of using a dimensional approach to estimate multiple, alternative pathways is the ability to map how households occupy hybrid spaces across several dimensions, in these cases potentially engaging to varying degrees with both cash and agricultural economies. Indeed, the many Kenyan households in the upper right hand corner of Fig 1a show that households can often occupy hybrid spaces that combine more than one avenue for advancement. Meanwhile, such opportunities seem less available in Ethiopia indicated by the large gaps in the 2-dimensional space (Fig 1e). This approach also potentially provides a common metric for future work to trace how different groups within countries—e.g., defined by region, ethnolinguistic identity, or occupation—follow different hybrid trajectories over time, or how single households in longitudinal surveys progress differently through livelihood space over time [67–69]. Finally, the extraction of orthogonal dimensions with MCA improves on using raw asset ownership as a measure of economic achievement. Specifically, ownership of any asset can be both a proxy of advancement along one dimension and of deprivation along the other. For example, owning more cows in Nepal is correlated with higher values on the agricultural dimension (r = 0.35, Table 1), but negatively correlated with the cash economy dimension (r = -0.45). Thus, in this case, using raw number of animals as a measure would confound success in the agricultural economy and lack of success in the cash economy, and could lead to very different conclusions. Indeed, in Nepal, we find that doubling the number of cows owned is associated with a significant *decline* of 0.7 kg/m2 in adult BMI (95% CI = (-1.0,-0.4)), because cows considered in isolation are a proxy of both success in one domain and lack of success in another [54]. By contrast, MCA identifies independent dimensions of achievement in the cash and agricultural economy that are both positively associated with adult BMI (Fig 3), indicating both dimensions are associated with increased energy reserves.

Although these additional dimensions are reliable and are related to substantial increases in food security and physical growth, they also point to a number of distinctions between success in cash and agricultural economies that are worth pursuing in future analyses. For example, the effect of the first cash economy dimension is consistently high on all three measures of nutrition and growth—food security, adult BMI, and child growth [39,70], indicating that it is a useful first approximation to economic advancement in contemporary low-income settings. By contrast, the relationship of the second agricultural economy dimension with these same measures shows striking variability between countries, from highs in Kenya and Nepal to lows in Ethiopia where agricultural success appears to have negligible associations with growth. This variability raises important questions about how local social and economic factors can create opportunities and barriers to translating agricultural success into better nutrition and growth. For example, why does agricultural achievement have stronger relationships with physical growth in some settings (Kenya) compared to other settings (Ethiopia)? Does this reflect the different opportunities for exchanging the fruits of agricultural success into human growth? Does it reflect ecological factors that might result from increasing engagement in the agricultural economy, such as higher burdens of infectious disease, which can compromise growth? Or does it arise from different meanings of a standard deviation increase along the agricultural dimension? For example, if there is less inequality in the agricultural economy than in the cash economy in a specific country, a standard deviation increase along the agricultural dimension 2 would indicate a smaller change in material conditions than a standard deviation increase along the cash economy dimension. If this were the case, one standard deviation increase in the agricultural economy would have a smaller association with key outcomes, such as physical growth. Future efforts to calibrate these dimensions with other measures of household economic capacity, such as caloric intake, consumption expenditures or total net worth, should permit future comparison achievement along these different dimensions across surveys and countries [22].

Here we have focused on demonstrating the reliability, interpretability and construct validity of alternative dimensions of material wealth. We also show that at least one additional second dimension captures success in the agricultural economy and is independently associated with physical growth and food security in adults and children. Although this expands on existing one-dimensional models, it still leaves room for further refinements. Closer inspection of any specific context will likely reveal subtle distinctions in activities—petty trading, service oriented activities, transport, salaried employment, agricultural wage labor—that go beyond the two coarse-grained dimensions examined here. In such cases, locally grounded ethnography could assist in identifying the diversity of livelihood activities and to determine other kinds of assets and activities that could be measured to assess these additional dimensions of economic activity [52]. In addition, we have focused here on identifying key dimensions of material wealth from asset data. However, researchers interested in categories of household production rather than dimensions of wealth accumulation may choose other kinds of data reduction techniques, such as k-means clustering that identify classes of households rather than dimensions along which households might vary [71].

Despite these limitations, we expect that estimates of success along these alternative pathways to prosperity will permit researchers to examine how achievement along different pathways can shape a wide range of risks, constraints, and life chances for individuals and households. For example, when are there trade-offs in livelihoods, such that success along one dimension restricts success along other dimensions? How do these different dimensions of wealth relate to other key indicators of human development and well-being—such as infectious disease risk, life satisfaction, and mental health [72]? Is heightened risk of HIV among “wealthier” households in many parts of sub-Saharan Africa an effect of increased wealth in general, or rather the kinds of far-flung social interactions that arise with one kind of wealth—success in the cash economy [44]? What kinds of livelihood success are most at risk in the face of different kinds of economic and environmental shocks [57,73]? Does the commonly found negative relationship between wealth and fertility change when assessed using different dimensions of wealth [4,55,56]? Future work on these and related questions will hopefully yield further insights into the affordances and risks created by different forms of economic achievement in low-income settings. In addition to permitting researchers to tackle new questions about the role of material wealth in well-being and development, multidimensional estimates of wealth may also provide policymakers new avenues: (1) for identifying and evaluating efforts to reduce inequities in access to basic services [74], (2) for targeting those households and individuals for interventions that are most deprived along multiple dimensions, and (3) for developing and evaluating interventions that foster opportunities along multiple pathways to economic improvement [52].

Across the six countries considered here, the first two dimensions of variation in livelihood space appear to reflect directional achievement as proxied by food security and growth. However, there is no a priori reason why all dimensions should have a clear directional interpretation. Indeed, the third dimensions appear to capture different kinds of agricultural accumulation characteristic of different ecological zones. For example, one pole of the third dimension in Kenya reflects households living in highland areas with cows and chickens, while the other pole reflects households in lowland semi-arid areas that are more likely to own goats, sheep, and donkeys. Although these do not have a clear directional interpretation, our findings show that these different trajectories of accumulation can still have associations with nutrition and physical growth.

These findings also have several implications for the current and future use of asset-based wealth measures. First, current studies using single-dimensional models of material wealth should in most cases be interpreted as examining one specific kind of success—advancement in a cash economy [44]. Second, hundreds of surveys already contain the kinds of variables we have analysed here, providing the opportunity to examine these additional dimensions of material wealth without collecting new data. Third, while the current set of assets is sufficient to estimate pathways of success in agricultural economies, the items in most surveys still appear to be biased to success in the cash economy and they likely still miss locally relevant assets that provide important information about local livelihoods. As an example, systematic collection of data on livestock in the demographic and health surveys only began about a decade ago. Before those were added to surveys, it would have been challenging to estimate reliable dimensions reflecting success in agricultural economies. Indeed, the lower internal reliabilities of the second dimension compared to the first dimension in the current analyses may partially be an artifact of implicit bias in current surveys toward proxies for success in the cash economy. Locally grounded work aimed at improving the set of agricultural indicators (and other livelihood indicators) would provide more reliable estimates of success in alternative economies.

Our findings from six countries across three world regions show that currently popular one-dimensional wealth capture an important pathway of economic achievement through cash economies, but simultaneously miss alternate agricultural pathways to success in low-income countries. That is, in each country, there are material *wealths* rather than a single kind of material wealth. We demonstrate how a multi-dimensional model applied to existing data can estimate additional reliable dimensions that: (1) capture these locally meaningful pathways to prosperity and (2) provide additional information about food security and physical growth. In addition to creating new opportunities for exploring the role of inequality in human development and well-being, these analyses and findings also highlight the value of on-the-ground research when developing such measures. For example, agricultural economies can differ markedly across countries and regions, and locally grounded fieldwork can provide important insights into what additional assets and livelihood pathways are available and valuable in specific cultural settings. Moreover, an understanding of local ecological and livelihood diversity can assist researchers in understanding why and how specific assets would be associated with different livelihood dimensions and how to interpret finer-grained dimensions of variation. Such efforts should contribute to improved, multidimensional models of wealth inequality that can help us understand how diverse pathways of economic advancement differentially shape individual risks and opportunities.

## Supporting information

S1 Fig Rural Nepal households (a; n = 9280) and Guatemalan households (b; n = 3852) mapped along two dimensions of agricultural livelihood. (DOCX)

S1 File Interpreting dimension 3 in Nepal and Guatemala. (DOCX)

S1 Table Sample sizes for the six country surveys. (DOCX)

S2 Table Frequencies of items by survey. (DOCX)

S3 Table Increase in food security and physical growth with 1 SD increase in livelihood dimension. (DOCX)
