# Supplementary material for: Material wealth in 3D: Mapping multiple paths to prosperity in low- and middle- income countries
Source: PLoS One. 2017 Sep 8;12(9):e0184616. doi: 10.1371/journal.pone.0184616 (PMC5590995; doi:10.1371/journal.pone.0184616)
Supplement: S1 Fig — (DOCX) [file pone.0184616.s001.docx]

**Supplementary Materials.**

**Figure S1. Rural Nepal households (a; n =9280) and Guatemalan households (b; n = 3852) mapped along two dimensions of agricultural livelihood.** Dim2 = 2^nd^ MCA dimension, Dim3 = 3^rd^ MCA dimension. Individual gray dot = household. Arrow starts = centroid of households without the specified animal. Arrow end = centroid of households having achieved a certain number of animals. Black dots = centroid of households with specified characteristic.

**
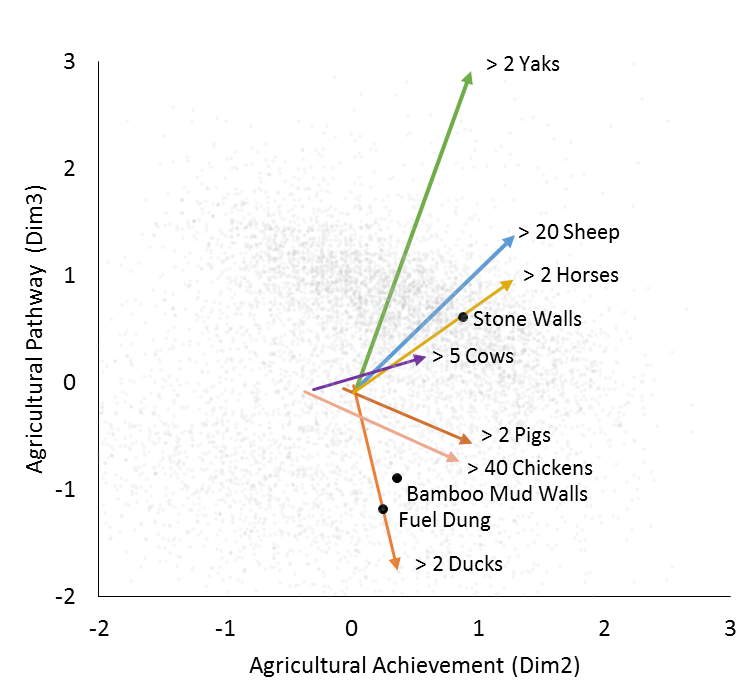
**

**
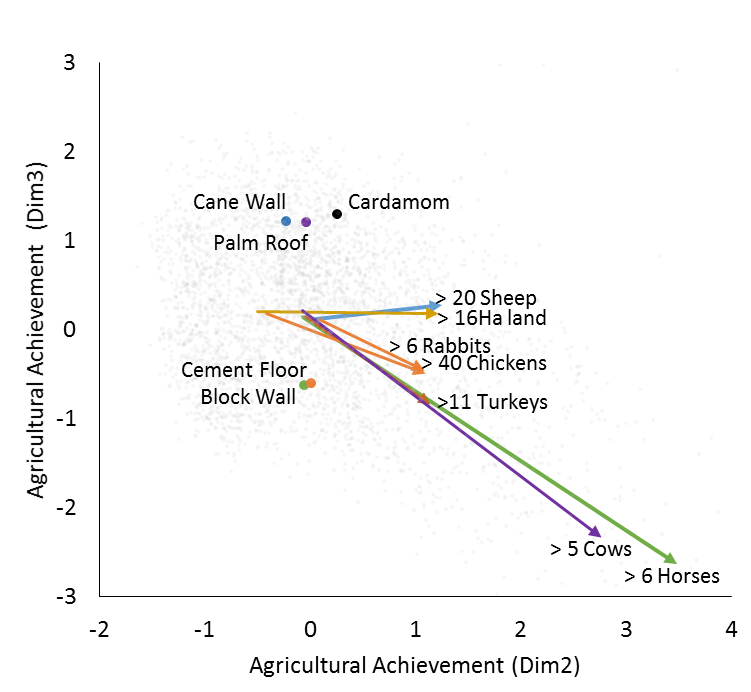
**
