# Supplementary material for: Material wealth in 3D: Mapping multiple paths to prosperity in low- and middle- income countries
Source: PLoS One. 2017 Sep 8;12(9):e0184616. doi: 10.1371/journal.pone.0184616 (PMC5590995; doi:10.1371/journal.pone.0184616)
Supplement: S1 File — (DOCX) [file pone.0184616.s002.docx]

**Supplementary Materials.**

***Interpreting Dimension 3 in Nepal and Guatemala.***

As in Kenya, the third dimension in Nepal reflects variation in ecological zone, as well as housing construction unique to different zones. Most notably, increases in dimension 2 are positively correlated with higher altitude (r = 0.64, p < 0.001) as well as housing characteristics (stone wall r = 0.52, public tap r = 0.36, p < 0.001) and to a lesser extent livestock (yak r = 0.10, horses r = 0.11, and sheep r = 0.08, p < 0.001) more common at higher altitudes. By contrast, lower values on dimension 2 are correlated with housing characteristics (bamboo mud wall r = -0.49, tubewell r = -0.68, p < 0.001) and animals (ducks r = -0.27, p < 0.001) that are common at lower altitudes (SM Figure S1a). Similarly, dimension 3 in Guatemala reflects regional variation in housing construction and livelihood. For example, values on dimension 3 are highest in northern mountainous frontier regions (Northern Highland mean=0.78, Petén mean=0.72) and lowest in regions closest to the capital city (Central mean = -0.27, Southeast = -0.18, and Metropolitan mean = -0.15). High values on dimension 3 are also associated with housing construction (Cane wall r = 0.39, palm roof r = 0.37, dirt floor r = 0.59, p < 0.001) and water sources (river water r = 0.30, p < 0.001) characteristic of frontier zones. Meanwhile, lower values are associated with housing characteristics (Block wall r = -0.46, cement floor = -0.53, electricity = -0.42, p < 0.001) and water sources (piped water r = -0.39, p < 0.001) common to areas more closely connect to the capital city. In addition to these housing differences, lower values on dimension 3 are also associated with owning more cows and horses (r = -0.38 & -0.15, p < 0.001) (See SM figure S1b).
