# Supplementary material for: Material wealth in 3D: Mapping multiple paths to prosperity in low- and middle- income countries
Source: PLoS One. 2017 Sep 8;12(9):e0184616. doi: 10.1371/journal.pone.0184616 (PMC5590995; doi:10.1371/journal.pone.0184616)
Supplement: S1 Table — (DOCX) [file pone.0184616.s003.docx]

**Supplementary Materials.**

**SM Table S1.** **Sample sizes for the six country surveys.**

|  | **Year** | **Households** | **Food Security** | **Women (20-49)** | **Children (0-24)** |
| --- | --- | --- | --- | --- | --- |
| Bangladesh | 2011 | 17141 |  | 13453 | 2070 |
| Nepal | 2011 | 10826 | 10826 | 3867 | 530 |
| Kenya | 2014 | 17409 | 17409 | 9980 | 4100 |
| Ethiopia | 2010 | 16702 |  | 10000 | 1779 |
| Tanzania | 2015 | 12563 | 12563 | 7801 | 1958 |
| Guatemala | 2000 | 7276 |  | 3866 | 1863 |
