# Supplementary material for: Material wealth in 3D: Mapping multiple paths to prosperity in low- and middle- income countries
Source: PLoS One. 2017 Sep 8;12(9):e0184616. doi: 10.1371/journal.pone.0184616 (PMC5590995; doi:10.1371/journal.pone.0184616)
Supplement: S2 Table — (DOCX) [file pone.0184616.s004.docx]

**Supplementary Materials.**

**SM Table S2. Frequencies of items by survey**

|  | Universal | Bangladesh 2011 | Ethiopia 2011 | Guatemala 2000 | Kenya 2014 | Nepal 2011 | Tanzania 2015 |
| --- | --- | --- | --- | --- | --- | --- | --- |
| Variable Number | Water Source |  |  |  |  |  |  |
| 1 | PipedDwelling | 5.7 | 1 | 57 | 7.2 | 5.5 | 3.2 |
| 2 | PipedYard | 4.5 | 10.1 | 13 | 20.5 | 16.9 | 8 |
| 3 | PublicTap | 2.1 | 23.3 | 3.9 | 15.7 | 24.5 | 15.5 |
| 4 | PipedNeighbor |  |  |  |  |  | 11.4 |
| 5 | Tubewell | 85.5 | 3.3 | 13.9 | 6.3 | 38.9 | 4.6 |
| 6 | ProtectedWell | 0.2 | 6.8 |  | 7.8 | 1.9 | 13.2 |
| 7 | UnprotectedWell | 0.2 | 3.6 |  | 5.7 | 2.1 | 16.8 |
| 8 | ProtectedSpring | 0 | 9 |  | 8.1 | 0.2 | 3 |
| 9 | UnprotectedSpring | 0 | 25.3 |  | 3.6 | 1 | 6.9 |
| 10 | River | 1.2 | 16.4 | 7 | 15.6 | 5 | 12.3 |
| 11 | Stonetap |  |  |  |  | 3.6 |  |
| 12 | Rainwater | 0.4 | 0.1 | 0.6 | 3.7 | 0 | 1.2 |
| 13 | TankerTruck | 0 | 0.1 | 0.4 | 0.5 | 0.7 | 1.6 |
| 14 | Cart | 0 |  |  | 1.2 |  | 0.6 |
| 15 | BottledWater | 0.1 | 0.1 |  | 1.9 | 0.9 | 1.6 |
| 16 | OtherWater | 0 | 1 | 4.2 | 1.8 | 0 | 0.1 |
|  | Toilet |  |  |  |  |  |  |
| 17 | FlushToPipe | 2.9 | 0.6 | 29.7 | 8.3 | 6.5 | 0.7 |
| 18 | FlushToSeptic | 7.9 | 0.6 | 3.7 | 6.3 | 36.7 | 2.6 |
| 19 | FlushtoPit | 1 | 1.6 |  | 2.2 | 4.4 | 13.4 |
| 20 | FlushToElsewhere | 2.4 | 0 | 43.6 | 0.1 | 0.1 | 0.3 |
| 21 | FlushDK | 2.2 | 0 |  | 0.4 | 0 | 0 |
| 22 | VIP | 12.4 | 1.7 |  | 15.3 | 0.9 | 2.7 |
| 23 | PitSlab | 28.3 | 8.8 |  | 20 | 8.3 | 16.1 |
| 24 | PitOpen | 31.3 | 43.5 |  | 36 | 7.1 | 14.9 |
| 25 | PitOpenNonWashable |  |  |  |  |  | 39.4 |
| 26 | NoFacility | 4.6 | 38.3 | 12.9 | 9.9 | 35.5 | 9.5 |
| 27 | Compostingtoilet | 0 | 4.5 |  | 0.4 | 0.2 | 0.1 |
| 28 | BucketToilet | 0 | 0 | 10.1 | 0.1 | 0 | 0 |
| 29 | HangingToilet | 6.9 | 0 |  | 0.4 | 0 | 0 |
| 30 | OtherToilet | 0 | 0.3 |  | 0.5 | 0 | 0.3 |
|  | Flooring |  |  |  |  |  |  |
| 31 | FloorEarth | 74.1 | 50.7 | 40.3 | 29.8 | 65.7 | 56.5 |
| 32 | FloorDung |  | 34 |  | 17.4 | 0.4 | 0.3 |
| 33 | FoorWood | 0.2 | 0.1 | 0.4 | 0.2 | 1.7 | 0 |
| 34 | FloorPalm | 0 | 0.7 |  | 0 | 0 | 0 |
| 35 | FloorParquet | 0 | 0.2 |  | 0.3 | 0.4 | 0 |
| 36 | FloorVinyl |  | 6.2 |  | 0.6 | 1.7 | 0.1 |
| 37 | FloorCeramic | 1.5 | 0.4 | 9.8 | 3.3 | 0.3 | 3.8 |
| 38 | FloorCement | 24.1 | 4.3 | 36.5 | 46.4 | 21.7 | 37.5 |
| 39 | FlorrCarpet | 0.1 | 2.6 |  | 1.9 | 8 | 1.5 |
| 40 | FloorOther | 0 | 0.7 | 13.1 | 0.1 | 0.1 | 0.1 |
|  | Wall |  |  |  |  |  |  |
| 41 | WallNone | 0 | 0.1 |  | 0.4 | 0 | 0 |
| 42 | WallGrass |  |  |  |  |  | 0.3 |
| 43 | WallCane | 6 | 8.1 | 6.7 | 1.1 | 2.1 | 2.8 |
| 44 | WallDirt | 12.8 |  |  | 32.3 | 6 |  |
| 45 | WallBambooMud | 9.3 | 75.7 |  | 3.4 | 19.8 | 17.4 |
| 46 | WallStoneMud | 0 | 8.6 | 2.8 | 1.2 | 27.9 | 0.9 |
| 47 | WallAdobe |  | 0 | 27.3 | 0.2 |  |  |
| 48 | WallPlywood |  | 0 |  | 0.3 | 0.8 |  |
| 49 | WallCardboard | 0 | 0 |  | 0 | 0 |  |
| 50 | WallReusedWood |  |  |  | 1.1 | 0.4 |  |
| 51 | WallTin | 42.8 |  | 3 | 7.9 |  |  |
| 52 | WallCement | 21.4 | 1.1 | 1.4 | 10.9 | 28.6 | 4 |
| 53 | WallStoneLime | 0.6 | 1.8 |  | 19.9 | 1.2 |  |
| 54 | WallMudBricks |  |  |  |  |  | 23.6 |
| 55 | WallBricks | 5.5 | 0.2 | 3.7 | 7.2 | 7.1 | 30.2 |
| 56 | WallCementBlock |  | 1.5 | 38.7 | 3.6 | 1.1 | 17.8 |
| 57 | WallCoveredAdobe |  | 0.1 |  | 0.5 |  |  |
| 58 | WallWood | 1.4 | 0.9 | 15.7 | 8.9 | 4.5 | 0.7 |
| 59 | WallOther | 0.2 | 1.9 | 0.9 | 1 | 0.5 | 1.1 |
|  | Roofing |  |  |  |  |  |  |
| 60 | RoofNone | 0.1 |  |  |  | 0.1 | 0.1 |
| 61 | RoofThatch | 3 | 48.5 | 5.5 | 10 | 17.9 | 22.8 |
| 62 | RoofDung |  |  |  | 0.6 |  |  |
| 63 | RoofMat |  | 0.7 |  |  | 0 | 1.8 |
| 64 | RoofBamboo | 0.1 | 1.4 |  |  | 0.2 | 0.1 |
| 65 | RoofWoodPlank | 0 | 0.9 |  |  | 0.8 | 0 |
| 66 | RoofCardboard | 0 |  |  |  | 0 |  |
| 67 | RoofTin | 85.1 | 46.8 | 70.1 | 84.5 | 29.9 | 74.2 |
| 68 | RoofTinCans |  |  |  | 0.3 |  |  |
| 69 | RoofWood | 0.1 | 0.1 |  |  | 0.2 |  |
| 70 | RoofCalamine |  | 0.1 |  | 0.7 | 1.7 |  |
| 71 | RoofCeramic | 0.2 |  | 11.7 |  | 26.3 |  |
| 72 | RoofCement | 10.4 | 0.2 | 2.2 | 2.5 | 22.2 | 0.4 |
| 73 | RoofShingles | 1 | 0 | 0.4 | 0.8 | 0 | 0.5 |
| 74 | RoofConcrete |  |  | 10 |  |  |  |
| 75 | RoofOther | 0.1 | 1.3 | 0.2 | 0.6 | 0.7 | 0.1 |
|  | Fueling |  |  |  |  |  |  |
| 76 | FuelElec | 0.1 | 0.7 | 3.3 | 0.4 | 0.1 | 0.5 |
| 77 | FuelLPG | 1.1 | 0.2 |  | 10.7 | 20.3 | 0 |
| 78 | FuelNatGas | 11.9 |  | 44.9 |  | 0 | 0 |
| 79 | FuelBioGas | 0.2 | 0.1 |  | 0.3 | 3.2 | 0 |
| 80 | FuelKerosene | 0.2 | 2.4 | 5.2 | 12.3 | 0.5 | 1.9 |
| 81 | FuelCoal | 0 |  | 10.5 | 0.1 | 0 | 0 |
| 82 | FueleCharcoal | 0.2 | 7.7 |  | 16.9 | 0.2 | 27.2 |
| 83 | FuelWood | 44.6 | 77 | 76.2 | 56.6 | 66 | 66.3 |
| 84 | FuelStraw | 1.2 |  |  | 1.2 | 3.2 | 0.1 |
| 85 | FuelAgricCrop | 31.7 |  |  | 0 | 0.9 | 0 |
| 86 | FuelDung | 8.7 | 7 |  | 0 | 4.8 | 0 |
| 87 | FuelNoCook | 0 | 1.8 |  | 1.5 | 0.7 | 1.1 |
| 88 | FuelOther | 0.2 | 0.3 |  | 0 | 0 | 0 |
|  | Livestock |  |  |  |  |  |  |
| 89 | Cattle 0 |  |  |  | 70.3 |  | 82 |
| 90 | Cattle 1 to 2 |  |  |  | 16.1 |  | 5.3 |
| 91 | Cattle 3 to 5 |  |  |  | 9 |  | 4.8 |
| 92 | Cattle 6 to 10 |  |  |  | 2.9 |  | 3.7 |
| 93 | Cattle 11 to 20 |  |  |  | 1 |  | 2.3 |
| 94 | Cattle > 20 |  |  |  | 0.7 |  | 1.8 |
| 95 | Cows 0 | 62.8 | 37.6 | 93.2 | 88 | 60 | 96.7 |
| 96 | Cows 1 to 2 | 24.3 | 26.2 | 4.1 | 8.1 | 24.1 | 2.2 |
| 97 | Cows 3 to 5 | 11.1 | 24.6 | 1.2 | 3 | 12.8 | 0.8 |
| 98 | Cows 6 1o 10 | 1.6 | 9.5 | 0.7 | 0.7 | 2.9 | 0.3 |
| 99 | Cows > 10 | 0.2 | 2.1 | 0.8 | 0.2 | 0.2 | 0.1 |
| 100 | Horses 0 |  | 71.6 | 93 | 93.3 | 99.4 | 97.6 |
| 101 | Horses 1 to 2 |  | 25.4 | 6 | 5.1 | 0.4 | 1.5 |
| 102 | Horses 3 to 5 |  | 2.9 | 0.7 | 1.1 | 0.1 | 0.7 |
| 103 | Horses > 5 |  | 0.1 | 0.3 | 0.4 | 0.1 | 0.3 |
| 104 | Goats 0 | 76.4 | 78.5 | 98.9 | 26.2 | 52.9 | 78 |
| 105 | Goats 1 to 2 | 15.3 | 9.6 | 0.5 | 8 | 16 | 5.7 |
| 106 | Goats 3 to 5 | 7 | 7.1 | 0.4 | 8.8 | 19.2 | 7.4 |
| 107 | Goats 6 to 10 | 1.2 | 3.3 | 0.2 | 4.7 | 9.2 | 4.8 |
| 108 | Goats 11 to 20 | 0.1 | 1.1 | - | 2.7 | 2.2 | 2.7 |
| 109 | Goats > 20 | 0 | 0.5 | - | 1.9 | 0.6 | 1.3 |
| 110 | Sheep |  | 72.6 | 97.6 | 85.1 | 98.5 | 92 |
| 111 | Sheep 1 to 2 |  | 13.1 | 0.5 | 5.3 | 0.7 | 2.3 |
| 112 | Sheep 3 to 5 |  | 9.4 | 0.7 | 4.9 | 0.3 | 2.9 |
| 113 | Sheep 6 to 10 |  | 3.8 | 0.6 | 2.5 | 0.2 | 1.7 |
| 114 | Sheep 11 to 20 |  | 1 | 0.4 | 1.2 | 0.1 | 0.7 |
| 115 | Sheep > 20 |  | 0.1 | - | 1 | 0.2 | 0.3 |
| 116 | Chickens 0 | 41.1 | 51.5 | 57.2 | 47.6 | 59.1 | 50.4 |
| 117 | Chickens 1 to 2 | 14.3 | 17.8 | 3.4 | 8.6 | 9.5 | 8.5 |
| 118 | Chickens 3 to 5 | 16.5 | 16.7 | 8.2 | 16.8 | 11.1 | 12.6 |
| 119 | Chickens 6 to 10 | 16 | 10.4 | 13.9 | 15.3 | 10.7 | 14.4 |
| 120 | Chickens 11 to 20 | 9.6 | 3.3 | 11.3 | 8 | 7.5 | 9.4 |
| 121 | Chickens 21 to 40 | 2.2 | 0.4 | 4.6 | 2.3 | 1.6 | 3.4 |
| 122 | Chickens > 40 | 0.3 | 0.1 | 1.5 | 1 | 0.5 | 1 |
| 123 | Bulls_Buffalo 0 | 99.6 |  |  |  | 67.9 |  |
| 124 | Bulls_Buffalo 1 to 2 | 0.2 |  |  |  | 25.2 |  |
| 125 | Bulls_Buffalo > 2 | 0.1 |  |  |  | 6.8 |  |
| 126 | Ducks 0 |  |  | 92.1 |  | 97.1 |  |
| 127 | Ducks 1 to 2 |  |  | 2.5 |  | 1.5 |  |
| 128 | Ducks 3 to 5 |  |  | 2.1 |  | 1.5 |  |
| 129 | Ducks > 6 |  |  | 2 |  |  |  |
| 130 | Pigs 0 |  |  | 83.1 |  | 88.7 |  |
| 131 | Pigs 1 to 2 |  |  | 12.2 |  | 9.5 |  |
| 132 | Pigs 3-5 |  |  | 3.3 |  | 1.7 |  |
| 133 | Pigs > 5 |  |  | 1.4 |  | - |  |
| 134 | Yaks 0 |  |  |  |  | 99.8 |  |
| 135 | Yaks 1 to 2 |  |  |  |  | 0.1 |  |
| 136 | Yaks > 2 |  |  |  |  | 0.1 |  |
| 137 | Camels 0 |  | 98.6 |  |  |  |  |
| 138 | Camels 1 to 2 |  | 0.9 |  |  |  |  |
| 139 | Camels > 2 |  | 0.5 |  |  |  |  |
| 140 | Rabbits 0 |  |  | 98.6 |  |  |  |
| 141 | Rabbits 1 to 2 |  |  | 0.7 |  |  |  |
| 142 | Rabbits > 2 |  |  | 0.7 |  |  |  |
| 143 | Turkeys 0 |  |  | 90.8 |  |  |  |
| 144 | Turkeys 1 to 2 |  |  | 4 |  |  |  |
| 145 | Turkeys 3 to 5 |  |  | 3.1 |  |  |  |
| 146 | Turkeys 6 to 10 |  |  | 1.5 |  |  |  |
| 147 | Turkeys > 11 |  |  | 0.6 |  |  |  |
| 148 | Bees 0 |  |  | 99.8 |  |  |  |
| 149 | Bees > 1 |  |  | 0.2 |  |  |  |
|  | Household Goods and Services |  |  |  |  |  |  |
| 150 | Electricity | 59.6 | 23 | 72.9 | 35.8 | 76.3 | 22.5 |
| 151 | Radio | 8.2 | 40.5 | 25.6 | 67.5 | 50.3 | 51.8 |
| 152 | Television | 39.9 | 10.4 | 53.1 | 34.3 | 46.9 | 20.2 |
| 153 | Fridge | 13.5 | 3.7 | 28.3 | 5.7 | 10.6 | 8.7 |
| 154 | Landline | 2.1 | 4.5 | 15.5 | 0.4 | 9.5 | 0.6 |
| 155 | Bednet |  |  |  | 64.8 | 67.8 | 72.5 |
| 156 | Mobile | 78.4 | 24.7 | 8.1 | 85.7 | 74.7 | 78 |
| 157 | WatchClock |  | 39.9 |  | 18.9 |  | 22.6 |
| 158 | Table |  | 34.9 |  |  | 53 |  |
| 159 | Chair |  | 41.9 |  |  | 46.9 |  |
| 160 | Bed |  |  |  |  | 91.3 |  |
| 161 | Sofa |  |  |  |  | 13.7 |  |
| 162 | Cupboard |  |  |  |  | 42.5 |  |
| 163 | Computer |  |  | 3.7 |  | 7.6 | 3.9 |
| 164 | Clock |  |  |  |  | 43.7 |  |
| 165 | Fan | 52.4 |  | 1.2 |  | 37.7 |  |
| 166 | Dhiki_Janto |  |  |  |  | 35.5 |  |
| 167 | Wardrobe | 35.2 |  |  |  |  |  |
| 168 | DVD | 12.1 |  |  |  |  |  |
| 169 | Water Pump | 5.8 |  | 1.5 |  |  |  |
| 170 | Mitad |  | 4 |  |  |  |  |
| 171 | KeroseneLamp |  | 14.3 |  |  |  |  |
| 172 | Solar |  |  |  | 10 |  |  |
| 173 | Stove |  |  | 48.2 |  |  |  |
| 174 | Microwave |  |  | 7.4 |  |  |  |
| 175 | Oven |  |  | 1.9 |  |  |  |
| 176 | Electric coffee |  |  | 10.4 |  |  |  |
| 177 | Blender |  |  | 38.3 |  |  |  |
| 178 | Juicer |  |  | 5.5 |  |  |  |
| 179 | Mill |  |  | 12.8 |  |  |  |
| 180 | Printer |  |  | 3.2 |  |  |  |
| 181 | Camera |  |  | 14.6 |  |  |  |
| 182 | CD Player |  |  | 17.3 |  |  |  |
| 183 | Tape Recorder |  |  | 48.8 |  |  |  |
| 184 | Typewriter |  |  | 17.4 |  |  |  |
| 185 | Video Camera |  |  | 1.7 |  |  |  |
| 186 | VCR |  |  | 5.9 |  |  |  |
| 187 | Iron |  |  | 16.9 |  |  | 3.1 |
| 188 | Hand Iron |  |  | 48.6 |  |  |  |
| 189 | Washer |  |  | 14 |  |  |  |
| 190 | Dryer |  |  | 5.7 |  |  |  |
| 191 | Vacum |  |  | 12.9 |  |  |  |
| 192 | Sewing Machine |  |  | 1.2 |  |  |  |
| 193 | Battery |  |  |  |  |  | 24.4 |
|  | Household Types and Ownership |  |  |  |  |  |  |
| 194 | Dedicated Kitchen |  |  | 44.9 |  |  |  |
| 195 | Shared Kitchen |  |  | 96.3 |  |  |  |
| 196 | Kitchen Chimney |  |  | 16 |  |  |  |
| 197 | Home_Inheritated |  |  | 13 |  |  |  |
| 198 | Home_Right to own |  |  | 3.1 |  |  |  |
| 199 | Home_Transfer |  |  | 14.2 |  |  |  |
| 200 | Home_Other |  |  | 0.8 |  |  |  |
| 201 | Owns_Structure |  |  | 58.6 | 60.2 |  |  |
| 202 | Rents_Structure |  |  | 10.3 | 34.7 |  |  |
| 203 | Consent_Structure |  |  |  | 4.6 |  |  |
| 204 | Squat_Structure |  |  |  | 0.5 |  |  |
| 205 | Missing_Structure |  |  |  | 0.1 |  |  |
|  | Land ownership |  |  |  |  |  |  |
| 206 | Owns_Homestead | 94.4 |  | 34.7 |  |  |  |
| 207 | AgriLand 0 Ha | 53.4 | 26.9 | 65.3 | 34.4 | 32.4 | 37.4 |
| 208 | AgriLand >0 to 1 Ha | 10.3 | 20.4 | 21.4 | 44.6 | 23.7 | 14 |
| 209 | Agriland >1 to 2 Ha | 2.2 | 19.2 | 5.5 | 11.6 | 2.2 | 14.3 |
| 210 | Agriland > 2 to 4 Ha | 1.2 | 19.6 | 3.2 | 3.9 | 1.7 | 15.3 |
| 211 | Agriland > 4 to 8 Ha | 0.3 | 9.3 | 1.5 | 0.8 | 0.4 | 11.4 |
| 212 | Agriland > 8 to 16 Ha | 0.1 | 2.4 | 1 | 0.8 | 0.2 | 4.4 |
| 213 | Agriland > 16 Ha | 0.1 | 0.7 | 2 | 0.5 | 0.1 | 2.8 |
| 214 | Owns_Land |  |  |  | 57.8 |  |  |
| 215 | Rents_Land |  |  |  | 31.8 |  |  |
| 216 | Consent_Land |  |  |  | 9.5 |  |  |
| 217 | Squat_Land |  |  |  | 0.9 |  |  |
| 218 | Missing_Land |  |  |  | 0.1 |  |  |
|  | Vehicles |  |  |  |  |  |  |
| 219 | Bicycle | 25.4 | 2.3 | 35 | 21.2 | 39.7 | 39 |
| 220 | Motorcycle | 5.4 | 0.2 | 3.8 | 7 | 10.9 | 9.2 |
| 221 | Car_Truck |  | 0.9 | 13.1 | 4.4 | 1.8 | 3.6 |
| 222 | Animal_Cart |  | 1 | 0.3 | 1.9 | 3 | 3.1 |
| 223 | BoatWMotor |  |  | 0.3 | 0.1 |  | 0.2 |
| 224 | Tempo/Autobike/CNG | 0.4 |  |  |  | 0.6 |  |
| 225 | Rickshaw/Van | 6.1 |  | 1.9 |  |  |  |
| 226 | Other truck |  |  | 0.7 |  |  |  |
|  | BUSINESS AND FINANCE |  |  |  |  |  |  |
| 227 | BankAccount | 27.2 | 10.5 | 18.1 | 47.5 | 62.5 | 39.9 |
| 228 | Owns Business |  |  | 36.4 |  |  |  |
| 229 | Owns Agricultural Business |  |  | 1.1 |  |  |  |
|  | Cultivation |  |  |  |  |  |  |
| 230 | Corn |  |  | 41.9 |  |  |  |
| 231 | Beans |  |  | 21.6 |  |  |  |
| 232 | Coffee |  |  | 9.4 |  |  |  |
| 233 | Cardamom |  |  | 2.2 |  |  |  |
| 234 | Potatos |  |  | 3.2 |  |  |  |
| 235 | Haba |  |  | 1.6 |  |  |  |
| 236 | Peas |  |  | 1.4 |  |  |  |
|  | Agricultural Equipment and Installations |  |  |  |  |  |  |
| 237 | Animal Equipment |  |  | 0.7 |  |  |  |
| 238 | Tractor |  |  | 0.2 |  |  |  |
| 239 | Tractor Equipment |  |  | 0.2 |  |  |  |
| 240 | Cultivator |  |  | 0.1 |  |  |  |
| 241 | Work Truck |  |  | 0.5 |  |  |  |
| 242 | Draft Animals |  |  | 4.5 |  |  |  |
| 243 | Fume Pump\ |  |  | 18.8 |  |  |  |
| 244 | Generator |  |  | 0.3 |  |  |  |
| 245 | Irrigation Equipment |  |  | 1.8 |  |  |  |
| 246 | Small Tools Misc |  |  | 44.1 |  |  |  |
| 247 | Coop |  |  | 4.6 |  |  |  |
| 248 | Mill |  |  | 1.1 |  |  |  |
| 249 | Silo |  |  | 4.6 |  |  |  |
| 250 | Dry Room |  |  | 0.5 |  |  |  |
|  | Totals | 115 | 123 | 159 | 140 | 146 | 130 |
