# Supplementary material for: Material wealth in 3D: Mapping multiple paths to prosperity in low- and middle- income countries
Source: PLoS One. 2017 Sep 8;12(9):e0184616. doi: 10.1371/journal.pone.0184616 (PMC5590995; doi:10.1371/journal.pone.0184616)
Supplement: S3 Table — (DOCX) [file pone.0184616.s005.docx]

**Supplementary Materials.**

**SM Table S3. Increase in food security and physical growth with 1 SD increase in livelihood dimension.** 95% CI in parentheses. Adjusted for age, education, and urban residence.

|  | Dimension | Food Security  (SD change) | Female BMI  (kg/m^2^ change) | Height-for-Age  (Z-score change) |
| --- | --- | --- | --- | --- |
| Nepal | 1 | 0.41 (0.37,0.44) | 1.8 (1.5,2.1) | 0.38 (0.21,0.54) |
|  | 2 | 0.31 (0.25,0.35) | 0.7 (0.4,1.0) | 0.23 (0.08,0.37) |
| Bangladesh | 1 |  | 1.6 (1.4,1.9) | 0.25 (0.16,0.35) |
|  | 2 |  | 0.4 (0.2,0.5) | 0.11 (0.04,0.19) |
| Kenya | 1 | 0.20 (0.16,0.23) | 2.1 (1.6,2.5) | 0.16 (0.04,0.27) |
|  | 2 | 0.15 (0.13,0.18) | 1.0 (0.8,1.2) | 0.15 (0.07,0.22) |
| Ethiopia | 1 |  | 1.7 (1.2,2.2) | 0.28 (0.16,0.40) |
|  | 2 |  | 0.1 (-0.1,0.4) | 0.04 (-0.07,0.15) |
| Tanzania | 1 | 0.25 (0.18,0.32) | 2.6 (2.2,2.9) | 0.26 (0.13,0.40) |
|  | 2 | 0.24 (0.20,0.28) | 0.6 (0.4,0.8) | 0.12 (0.05,0.20) |
| Guatemala | 1 |  | 2.2 (1.6,2.9) | 0.48 (0.28, 0.69) |
|  | 2 |  | 0.5 (0.1, 1.0) | 0.03 (-0.08, 0.17) |
